# Canine platelets express functional Toll-like receptor-4: lipopolysaccharide-triggered platelet activation is dependent on adenosine diphosphate and thromboxane A2 in dogs

**DOI:** 10.1186/s12917-019-1997-3

**Published:** 2019-07-15

**Authors:** Ronald H. L. Li, Nghi Nguyen, Fern Tablin

**Affiliations:** 1 Department of Veterinary Surgical and Radiological Sciences, School of Veterinary Medicine, University of California, Davis, California USA; 2 Department of Anatomy, Physiology and Cell Biology, School of Veterinary Medicine, University of California, Davis, California USA

**Keywords:** Sepsis, Thrombosis, Platelet-priming, Inflammation, Aspirin, Pattern recognition receptor

## Abstract

**Background:**

Functional Toll-like receptor 4 (TLR4) has been characterized in human and murine platelets indicating that platelets play a role in inflammation and hemostasis during sepsis. It is unclear whether canine platelets could express functional TLR4 by responding to its ligand, lipopolysaccharide (LPS). We sought to determine if dogs express functional TLR4 and if LPS-induced platelet activation requires co-stimulation with ADP or thromboxane A_2_ (TxA_2_). Canine platelets were unstimulated (resting) or activated with thrombin or ADP prior to flow cytometric or microscopic analyses for TLR4 expression. We treated resting or ADP-primed platelets with LPS in the absence or presence of acetylsalicylic acid (ASA) and inhibited TLR4 with function blocking antibody or LPS from *Rhodobacter sphaeroides* (LPS-RS).

**Results:**

We discovered that dog platelets have variable TLR4 expression, which was upregulated following thrombin or ADP activation. LPS augmented P-selectin expression and thromboxane B_2_ secretion in ADP-primed platelets via TLR4. Inhibition of cyclooxygenase by ASA attenuated LPS-mediated P-selectin expression demonstrating that TLR4 signaling in platelets is partially dependent on TxA_2_ pathway.

**Conclusion:**

Expression of functional TLR4 on canine platelets may contribute to hypercoagulability in clinical septic dogs. Cyclooxygenase and TxA_2_ pathways in TLR4-mediated platelet activation may present novel therapeutic targets in dogs with sepsis.

## Background

Despite recent advances in veterinary medicine, mortality remains high in dogs with sepsis secondary to Gram negative bacterial infections [1–3]. One of the major components of the outer membrane of Gram negative bacteria is the endotoxin, lipopolysaccharide (LPS), whose receptor, Toll-like receptor 4 (TLR4), is present on the surface of a wide variety of immune cells such as dendritic cells, epithelial cells, polymorphonuclear cells and macrophages. Formation of the TLR4 receptor complex in response to LPS initiates signalling pathways leading to proinflammatory cytokine production and inflammatory response. In addition to being the primary effector cell in hemostasis, there is growing evidence demonstrating that platelets function as innate immune cells [4]. Bacteria like *E.coli* and *Streptococcus* directly interact with platelets leading to platelet activation and aggregation [5]. Murine and human platelets also express several Toll-like receptors (TLRs), suggesting that platelets can act as sentinel cells in detecting pathogen-associated molecular patterns (PAMPs) like LPS.

Thrombocytopenia, a common finding in septic dogs, is associated with mortality, though the exact mechanism of this hematologic abnormality is poorly understood [6–8]. Proposed mechanisms of sepsis-associated thrombocytopenia include decreased thrombopoiesis and increased platelet consumption and sequestration. Systemic platelet activation, which precedes platelet accumulation in organs and microvasculature in human septic patients, suggests that platelets may be the key effector for systemic coagulation during bacterial infection [9]. Systemic hypercoagulability could progress to disseminated intravascular coagulation, further impeding blood flow to tissues causing organ dysfunction. Andonegui et al. showed that platelet TLR4 is an important regulator of endotoxin-mediated thrombocytopenia in mice [10]. In another in vivo sepsis model, transfusion of TLR4 deficient platelets in platelet-depleted mice attenuated microvascular thrombosis [11]. These studies suggest that platelet TLR4 also may play a role in facilitating platelet activation in sepsis leading to microvascular thrombosis, and organ dysfunction in septic dogs. [10, 11] This, however, has never been demonstrated in this species. In one of the few canine studies, Yilmaz et al., demonstrated increased platelet aggregation in a lethal endotoxin shock model [8]. Another study, however, found that circulating platelets in dogs with septic peritonitis have decreased aggregation in response to multiple agonists [12].

The mechanism of platelet activation in sepsis has been extensively studied in mice and humans with conflicting results. While some investigators found that LPS stimulates human platelets to undergo activation and aggregation, others found that LPS does not directly stimulate platelets or that LPS-triggered activation requires synergistic stimulation by platelet agonists like ADP, collagen and thromboxane A_2_ (TxA_2_) [5, 13–16]. Because platelet activation mediated by TLR4 may account for the interplay between sepsis and thrombosis in dogs, a better understanding of platelet TLR4 expression and platelet response to LPS, is needed. We, therefore, aimed to examine platelet membrane TLR4 expression and determine if this expression is altered by the platelet agonists, ADP and thrombin. We also aimed to determine if LPS could activate platelets via TLR4. Specially, we sought to determine if LPS, in the absence or presence of ADP or TxA_2_, could stimulate platelet alpha-granule secretion. Lastly, we sought to determine if inhibition of platelet TLR4 could attenuate platelet response to LPS in the absence or presence of platelet priming by ADP.

## Results

Out of the 30 dogs studied, 14 dogs were female and 16 dogs were male. Age ranged from 0.33 to 13 years of age (mean 4.92). Of the 30 dogs, 19 were mixed breed dogs; 11 were purebreeds including 2 Labrador Retreivers, 5 Golden Retreivers, 1 Cataoula Hog Dog, 1 Weimaraner,1 Akbash Coban and 1 Bouvier des Flandres.

### Canine platelets express surface TLR4 and its expression is upregulated by thrombin and ADP

**Fig. 1:**
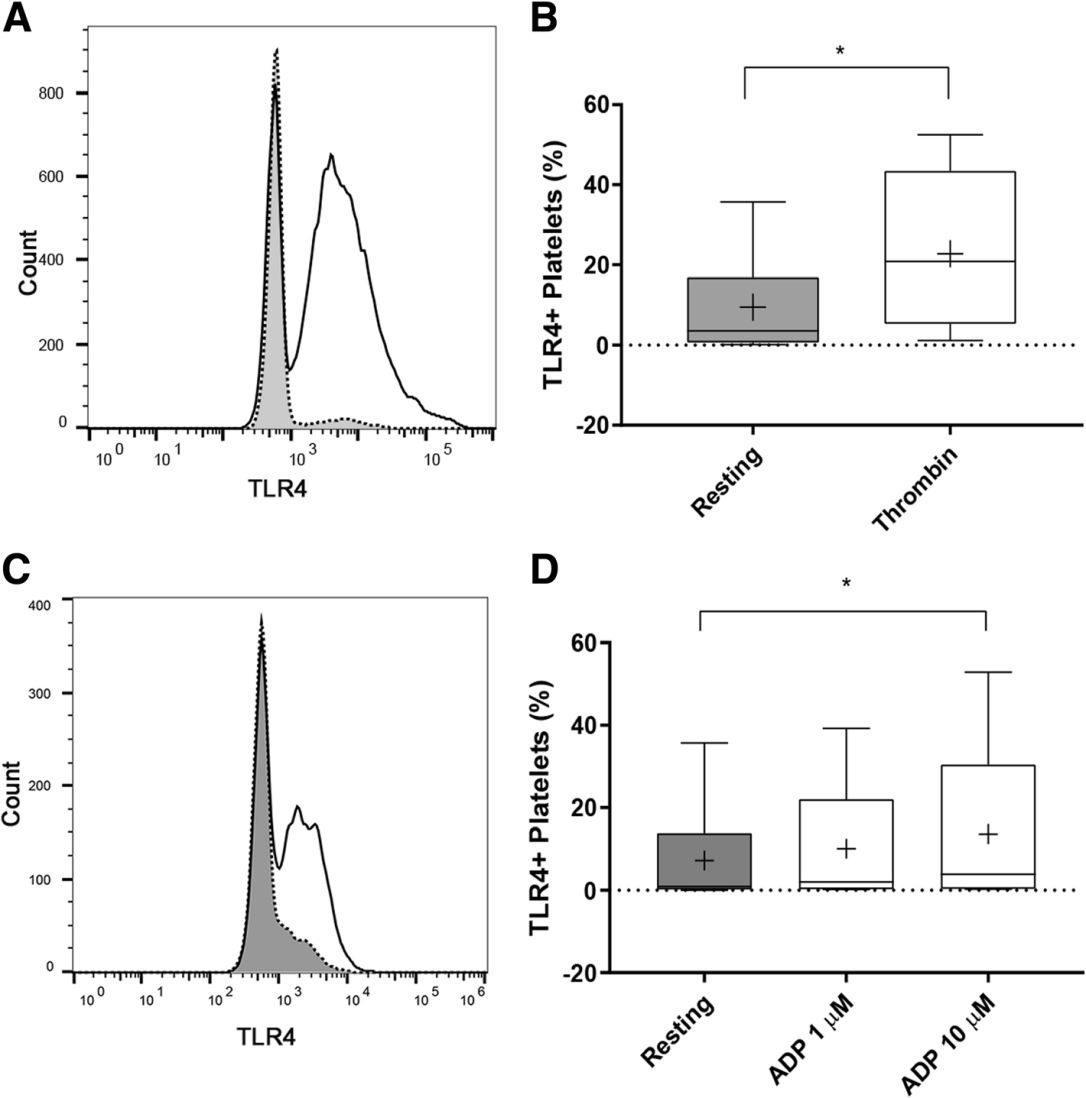
Platelet activation upregulates surface TLR4 expression. Platelet surface TLR4 expression was measured on isolated platelets from 10 dogs using flow cytometry. **a**,**c** Representative histograms of resting (unstimulated) platelets (grey) and activated platelets (clear) indicating increase in surface TLR4 expression following thrombin (**a**) and ADP (**c**) activation. **b** Thrombin stimulation led to significant increase in percentage (%) of TLR4-positive platelets compared to resting platelets. **d** Platelets stimulated with 10 μM ADP had significantly higher percentage of TLR-positive platelets. First and third quartiles were represented by the lower and upper boundaries, respectively. + and the line within the box represents the mean and median, respectively. Whiskers represent the range of data. * *p* < 0.05

**Fig. 2:**
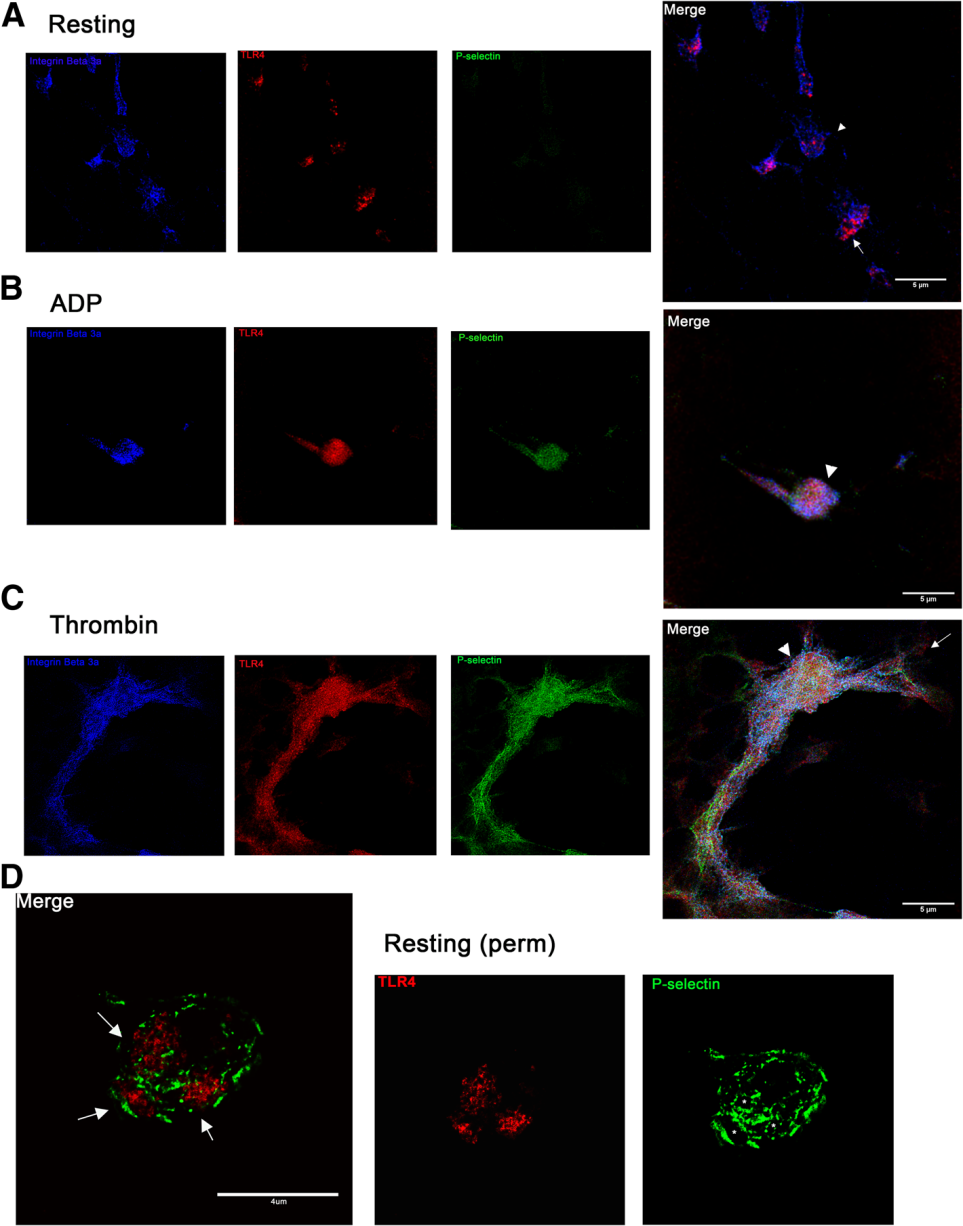
Representative confocal and super resolution immunofluorescence microscopy demonstrating TLR4 expression on **a** resting, **b** ADP-, **c** thrombin-activated and **d** permabilized resting canine platelets. **a-c** Following activation, platelets were fixed and stained for TLR4 (red), P-selectin (green) and the integrin β3 (blue). **a** In the absence of platelet agonists, resting platelets as outlined by the abundant integrin β3 show limited to no expression of P-selectin on the membrane surface. Note the aggregregated appearance of exteriorized TLR4 (arrow) **b**,**c** In ADP- and thrombin-activated platelets, TLR4 expression is upregulated and is evently distributed across the membrane surface. Note the formation of pseudopodia and colocalization of TLR4 and P-selectin (arrowheads) on activated platelets **c** Extensive platelet aggregation can be seen in thrombin-activated platelets. Scale bar = 5 μm. Original magnification 100x **d** Resting platelets were fixed, permabilized and stained for TLR4 (red) and P-selectin (green). A single z-plane is shown here to demonstrate the presence of alpha-granules within a platelet (asterisk). Intracellular TLR4 can be seen within the alpha-granules (arrows). Scale bar = 4 μm. Experiment was replicated twice from platelets isolated from 2 dogs

Resting platelets had a low surface expression of TLR4 (9.50%; IQR = 0.70–16.88) and the expression was highly variable among subjects with a coefficient of variation (CV) of 135.54%. Thrombin or 10 μM ADP significantly increased the number of TLR4-positive platelets relative to resting platelets (20.80%, IQR = 5.39–43.43, *p* = 0.0078; 12.12%, IQR = 1.31–45, *p* = 0.016, respectively) (Fig. 1a to d). CVs of TLR4 expression in thrombin- and ADP-stimulated platelets were 85 and 97.62%, respectively. ADP increased TLR4 expression in a dose-dependent manner (*p* = 0.047) (Fig. 1d). Compared to ADP-stimulated platelets, thrombin stimulation resulted in higher TLR4 MFI fold change (0.065 ± 0.064 vs. 0.14 ± 0.12), but this difference did not reach statistical significance (*p* = 0.078). Flow cytometry findings were confirmed by directly visualizing surface TLR4 expression using confocal and STED immunofluorescence microscopy. The platelet membrane was identified by detecting the highly expressed platelet integrin subunit, β3 (Fig. 2). As expected, resting platelets have minimal exteriorization of P-selectin and TLR4 was either sparcely expressed or expressed in clusters on the cell membrane of unpermeabilized platelets (Fig. 2a, arrow). Following ADP or thrombin-induced activation, surface expression of TLR4 and P-selectin was upregulated on the platelet membrane. Compared to the clustered conformation seen on resting platelets, TLR4 was distributed evenly on the membrane surface (Fig. 2b, c). In addition, co-localization of TLR4 and P-selectin on the membrane surface was detected on either ADP or thrombin-stimulated platelets (Fig. 2b, c). We then determined the location of intracellular TLR4 by permeabilizing fixed resting platelets prior to immunostaining. We found that TLR4 (Fig. 2d, Merge, arrows) was concentrated within the platelet alpha-granules as outlined by P-selectin (Fig. 2d, P-selectin, asterisks).

### LPS has a limited ability to stimulate alpha-granule secretion in canine platelets

**Fig. 3:**
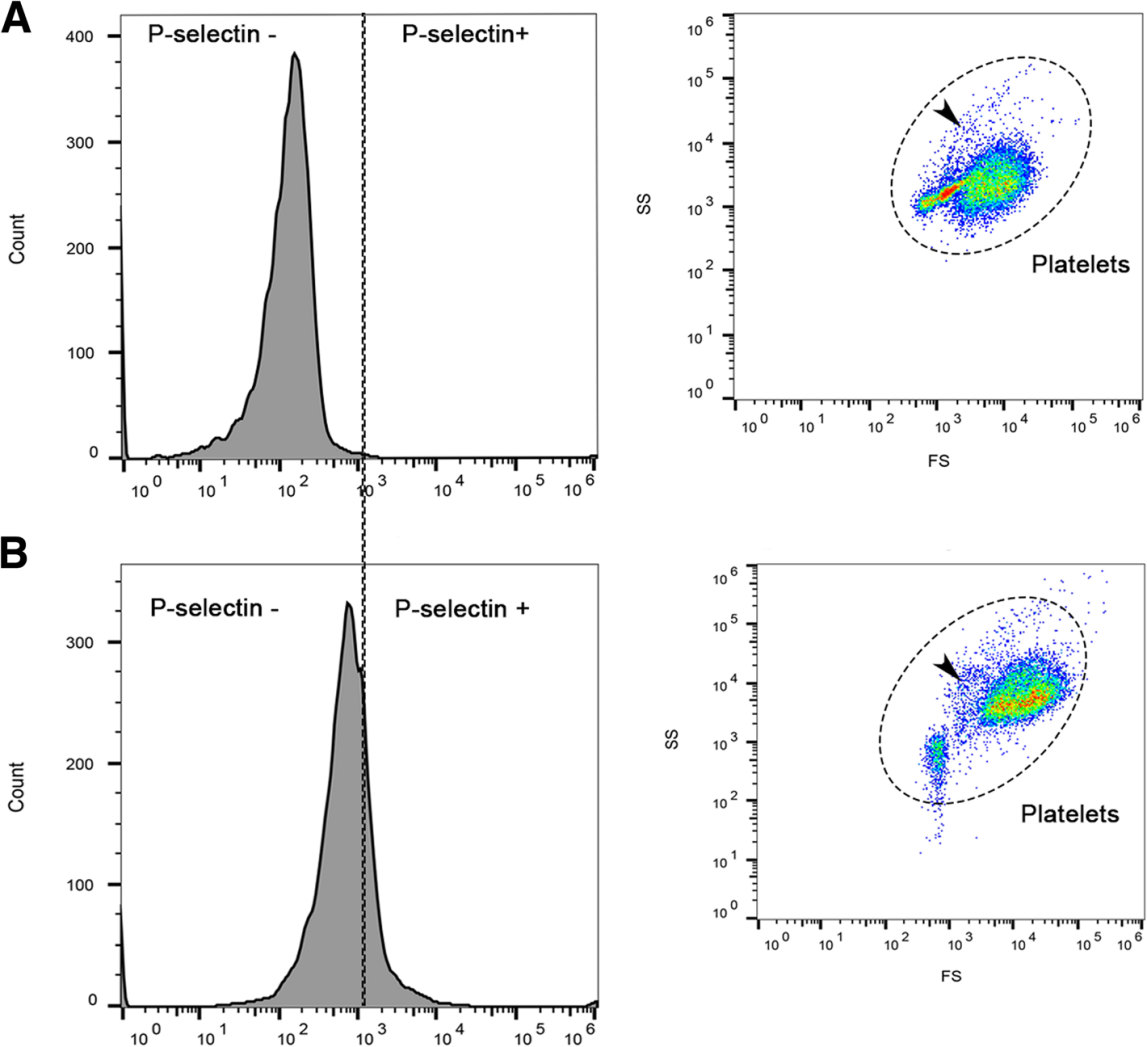
Variability of P-selectin expression on unstimulated/resting platelets. Representative histograms and scatter plots of flow cytometric analysis of unstimulated platelets isolated in 2 dogs on the same day. Platelets were identified by their light scatter properties and binding of CD61. Histogram demonstrates the number of activated platelets shown as P-selectin positive. **a** Resting platelets with mimimal P-selectin positive platelets and characteristic light scatter profile of canine platelets. **b** Unstimulated platelets collected from a different dog showed increased P-selectin-positive platelets with side-scatter property indicative of increased degranulation (arrow)

**Fig. 4:**
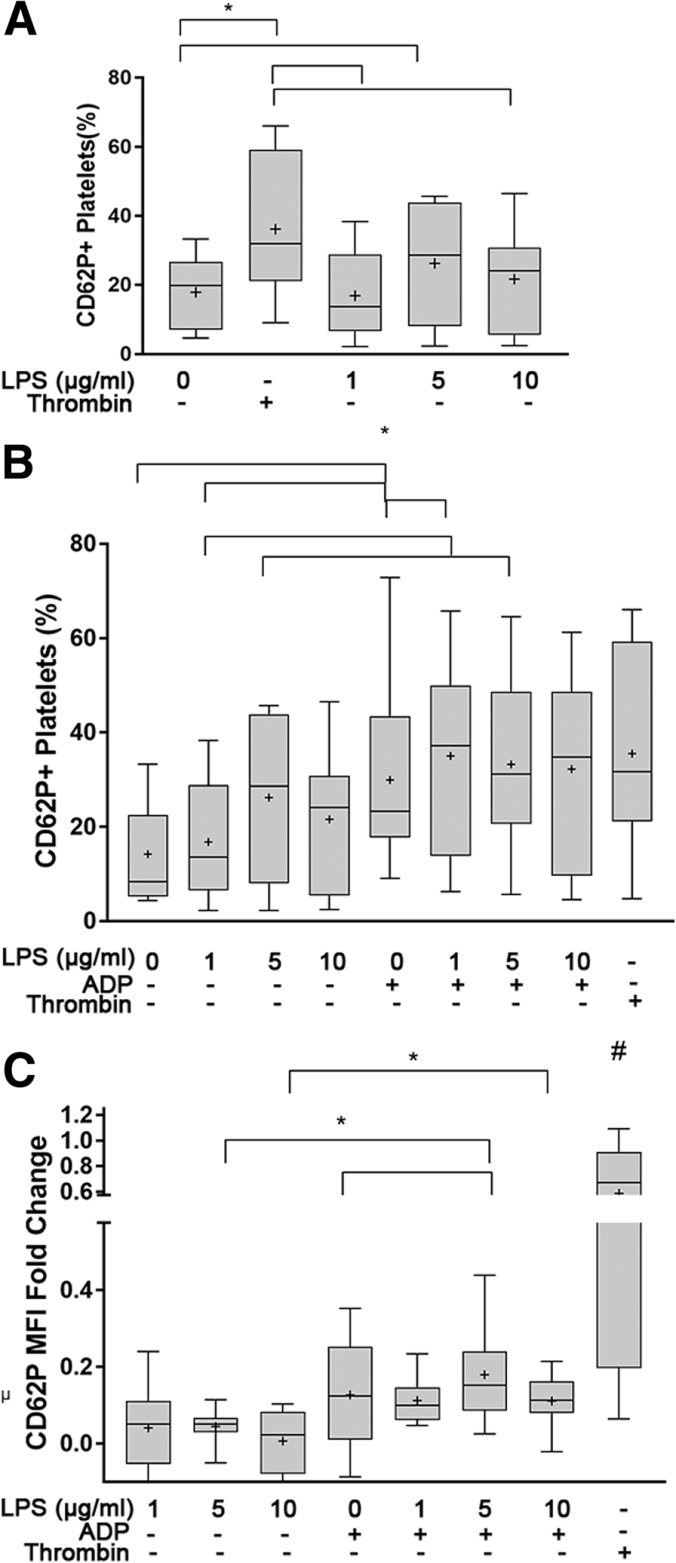
ADP-priming augments LPS-mediated alpha-granule secretion in canine platelets. Platelet alpha-granule secretion was assessed by surface P-selectin (CD62P) measured as percent (%) positive or mean fluorescence intensity (MFI) fold change on isolated platelets from 10 dogs using flow cytometry. Thrombin-stimulated platelets served as positive control. **a** Platelets were treated with 0, 1, 5, or 10 μg/ml LPS. Only platelets treated with 5 μg/mL LPS had significant increase in CD62P+ platelets compared to unstimulated platelets. **b**,**c** Platelets were primed with 10 μM ADP prior to stimulation with 0, 1, 5 or 10 μg/ml LPS. LPS stimulation with 1 μg/mL LPS in ADP-primed platelets significantly elevated the percentage of CD62P+ platelets **b** but did not increase MFI fold change **c** compared to ADP-primed platelets without LPS. LPS at 5 μg/mL significantly increase CD62P MFI fold change in ADP-primed platelets relative to those without LPS. ADP priming increased platelet response to LPS at 1 (**b**), 5 μg/mL (**b**,**c**) and 10 μg/mL (**c**). First and third quartiles were represented by the lower and upper boundaries, respectively. + and the line within the box represents the mean and median, respectively. Whiskers represent the range of data. * *p* < 0.05, # All treatments were significantly different (p < 0.05) from positive control

To determine if LPS could activate canine platelets, we measured surface P-selectin expression, using flow cytometry, as a marker of alpha-granule secretion following treatment with 0, 1, 5 or 10 μg/ml LPS. P-selectin expression on resting platelets was highly variable among dogs (Range 2 to 34%, CV = 73.59%) despite a standardized protocol (Fig. 3a,b). Although we could not find a dose-dependent effect of LPS on platelet P-selection expression (% P-selectin-positive platelets, *p* = 0.11; P-selectin MFI fold change, *p* = 0.38), 5 μg/ml LPS significantly elevated the number of P-selectin-positive platelets compared to resting platelets (26.23% ± 17.24 vs. 17.85% ± 10.53, *p* = 0.041) (Fig. 4a). This elevation was similar to surface P-selectin expression in thrombin-stimulated platelets (36.14% ± 20.85, *p* = 0.066). Treatment with either 1 or 10 μg/ml LPS did not significantly increase the number of P-selectin-positive platelets compared to resting platelets (16.82% ± 12.65, *p* = 0.74; 21.62% ± 15.4, *p* = 0.32, respectively) (Fig. 4a). No significant differences between P-selectin MFI fold change in platelets treated with 1, 5 or 10 μg/ml LPS (0.041 ± 0.11, 0.044 ± 0.047, 0.0064 ± 0.081, respectively, *p* > 0.05) were found. Compared to thrombin as an agonist (0.5873 ± 0.38), LPS stimulation resulted in significantly lower P-selectin MFI fold change (1 μg/ml, *p* = 0.0057; 5 μg/ml, *p* = 0.0045; 10 μg/ml, *p* = 0.0018) (Fig. 4c). Similarly, stimulation of platelets with all concentrations of LPS did not result in significant changes in the number of P-selectin-positive PDMV (*p* = 0.4833) nor MFI fold change (*p* = 0.22).

### Platelet priming by ADP potentiates LPS-mediated alpha-granule secretion

To determine if ADP could augment platelet response to LPS, we first primed platelets with 10 μM ADP, followed by 1, 5 or 10 μg/ml LPS. Compared to platelets treated with 5 μg/ml LPS alone, ADP priming prior to treatment with LPS of the same concentration resulted in significant elevation in P-selectin (20.51% ± 17.44 vs. 33.20% ± 19.83, *p* = 0.0032; MFI fold change 0.044 ± 0.047 vs. 0.18 ± 0.13, *p* = 0.0092) (Fig. 4b, c). ADP-priming prior to LPS stimulation also led to significant increase in P-selectin expression compared to platelets activated with ADP alone (MFI fold change: 0.18 ± 0.13 vs. 0.13 ± 0.14, *p* = 0.038) (Fig. 4c). When ADP-primed platelets were treated with 10 μg/ml LPS, P-selectin MFI fold was significantly higher compared to unprimed platelets treated with the same concentration of LPS (MFI fold change 0.11 ± 0.070 vs. 0.0064 ± 0.08, *p* = 0.00020) but this elevation did not differ from that in ADP-treated platelets (*p* = 0.059) (Fig. 4c). ADP-priming followed by LPS stimulation significantly increased the number of P-selectin-positive platelets relative to ADP-activated platelets (35.01% ± 20.48 vs 29.88% ± 19.70, *p* = 0.035) and unprimed platelets treated with 1 μg/ml LPS (35.01% ± 20.48 vs 16.82% ± 12.65, *p* = 0.013) (Fig. 4b).

TLR4 expression (MFI fold change) in resting platelets did not correlate with LPS-induced P-selectin expression (MFI fold change) in unprimed platelets (r = 0.25, r^2^ = 0.065, *p* = 0.51). Following ADP priming, however, TLR4 expression was positively and moderately correlated with LPS-mediated P-selectin expression (r = 0.70, r^2^ = 0.49, *p* = 0.036).

### ADP priming augments LPS-induced TxA_2_ synthesis

**Fig. 5:**
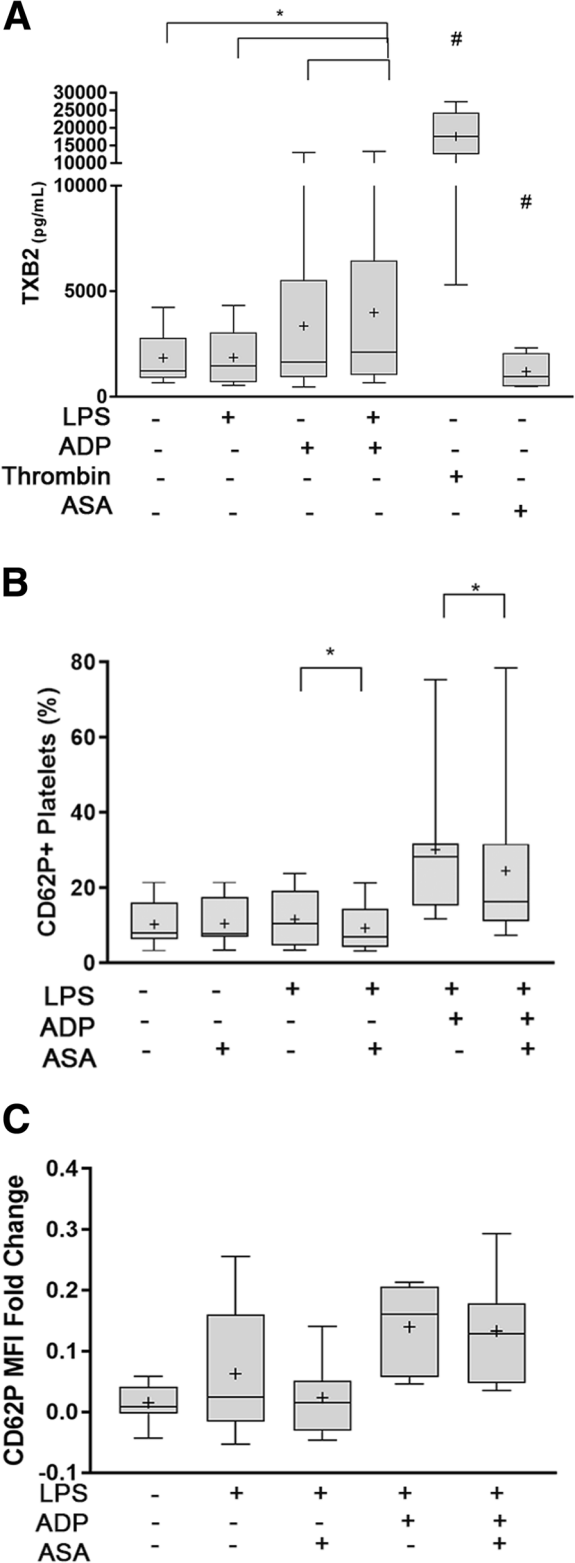
LPS amplifies ADP-mediated thromboxane B_2_ (TxB_2_) secretion and inhibition of platelet cyclooxygenase 1 attenuates LPS-mediated alpha-granule secretion. **a** Platelet TxB_2_ concentration was measured by ELSIA from platelet supernatant in 10 dogs. In the presence or absence of ADP, platelets were treated with 5 μg/ml LPS. Thrombin-stimulated and acetyl salicylic acid (ASA)-treated platelets served as positive and negative controls, respectively. LPS-treated platelets did not augment TxB_2_ production compared to unstimulated platelets and ADP-treated platelets. ADP priming in LPS-treated platelets led to more TxB_2_ than ADP-treated platelets, LPS-treated platelets and unstimulated platelets. **b**,**c** Isolated platelets from 10 dogs were pretreated with 100 μM acetylsalicyclic acid (ASA) prior to treatment with 5 μg/ml LPS with or without ADP. Platelet alpha-granule secretion was assessed by P-selectin (CD62P) measured as percent (%) positive **b** or mean fluorescence intensity (MFI) fold change **c** using flow cytometry. ASA significantly decreased the % P-selectin positive platelets **a** but not P-selectin MFI fold change **b** in LPS-treated platelets in the absence or presence of ADP-priming. First and third quartiles were represented by the lower and upper boundaries, respectively, and the line within the box represents the median. + represents the mean. Whiskers represent the range of date. * *p* < 0.05, # Significance between all treatments and controls (*p<0.05*)

To examine if LPS augments platelet TxA_2_ synthesis in the presence or absence of ADP, we measured TxB_2_ concentrations in platelet supernatant using ELISA. Using ASA-treated platelets as negative controls, we found that LPS did not significantly elevate TxB_2_ secretion relative to ASA-treated platelets (1460 pg/ml, IQR: 695.4–3048 vs. 952 pg/ml, IQR: 495.3–2062, *p* = 0.06). TxB_2_ concentration in LPS-treated platelets also was similar to that in resting platelets (1460 pg/ml, IQR: 695.4–3048 vs. 1839 pg/ml, IQR: 887.1–2788, *p* > 0.99). Following priming with ADP, LPS resulted in significant elevation in TxB_2_ secretion (3988 pg/ml IQR: 1042–6459) compared to resting platelets (*p* = 0.0020) and AA-reated platelets (*p* = 0.005). ADP-priming prior to LPS treatment also significantly increased TxB_2_ secretion compared to stimulation with LPS (*p* = 0.0039) or ADP (1637 pg/ml, IQR: 921.3–5530, p = 0.0039) alone (Fig. 5a).

### LPS-mediated alpha granule secretion requires TxA_2_

To determine whether TxA_2_ was required for LPS-mediated alpha granule secretion, we first treated platelets with ASA prior to LPS stimulation in the absence or presence of ADP. We found that ASA did not significantly affect the number of P-selectin-positive platelets in resting (ASA: 8.06%, IQR:6.38–16.12 vs. Resting: 7.87%, IQR:6.94–17.54, *p* = 0.84) (Fig. 5b) or ADP-stimulated platelets (ASA + ADP: 23.85%, IQR:16.53–36.18 vs. ADP: 32.25%, IQR:18.00–44.50, *p* = 0.10) . ASA also did not affect P-selectin MFI fold change in platelets activated with LPS (ASA + LPS: 0.014, IQR: − 0.043 – 0.045 vs. LPS:0.015, IQR: − 0.042 – 0.14, *p* = 0.37) or ADP (ASA + ADP: 0.15, IQR: 0.083–0.25 vs. ADP: 0.13, IQR: 0.097–0.29, *p* = 0.74) (Fig. 5c).

In unprimed platelets treated with LPS, number of P-selectin positive platelets was significantly lower in those with ASA treatment compared to those without (10.54%, IQR: 4.72–19.33 vs 6.97%, IQR: 4.15–14.45, *p* = 0.031). Inhibition of TxA_2_ synthesis by ASA also significantly attenuated the number of P-selectin positive platelets in ADP-primed platelets treated with LPS (16.35%, IQR: 11.03–31.63 vs. 28.15%, IQR: 15.33–31.68, *p* = 0.05) but not in P-selectin MFI fold change (0.13 ± 0.086 vs. 0.16 ± 0.071, *p* = 0.83) (Fig. 5b,c).

### LPS-mediated alpha-granule secretion is dependent on TLR4

**Fig. 6:**
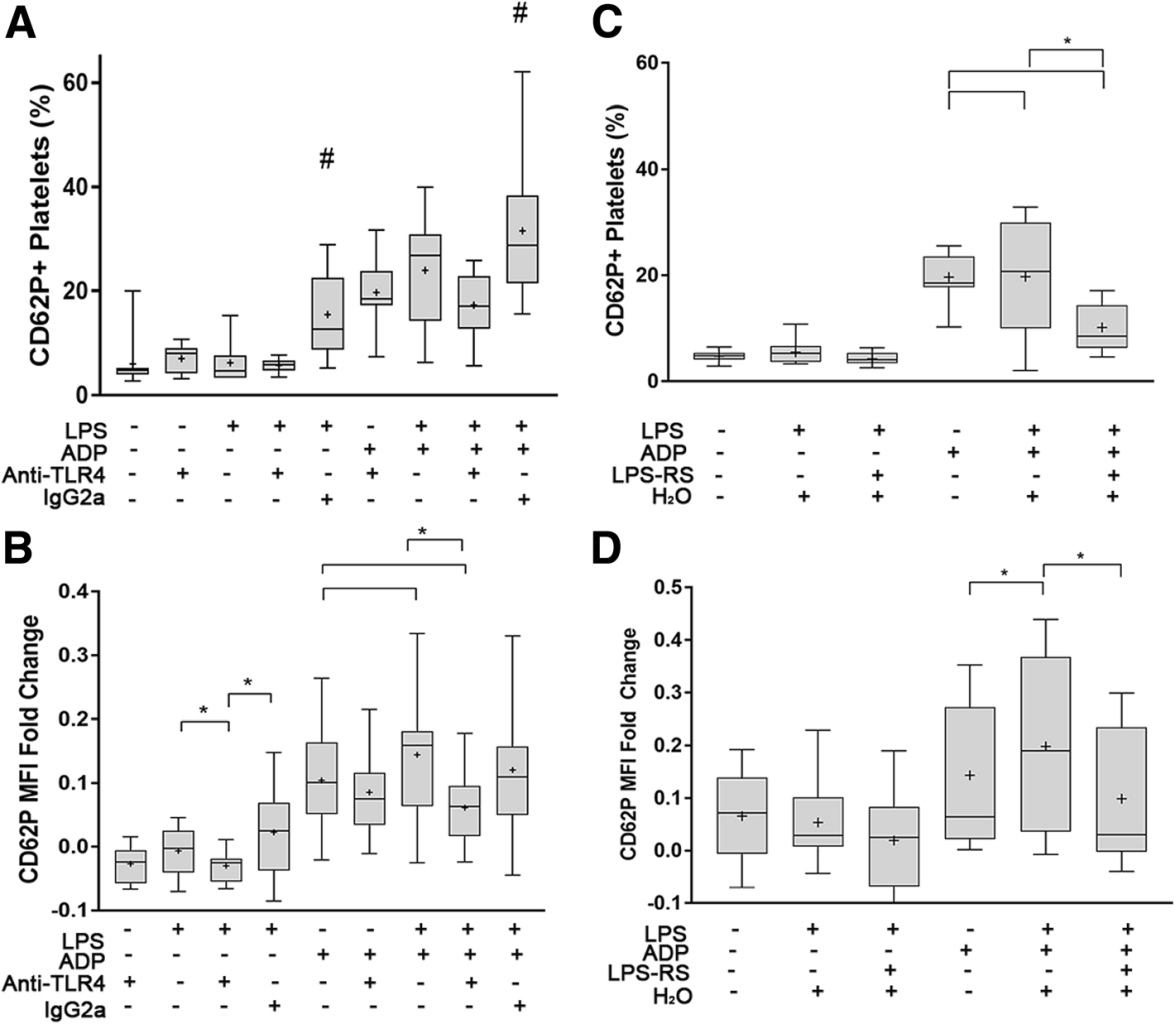
LPS-mediated alpha-granule secretion is dependent on platelet TLR4. Platelet P-selectin (CD62P) measured as percent (%) positive platelets or mean fluorescence intensity (MFI) fold change on isolated platelets (1 × 10^7^/ml) from 10 dogs using flow cytometry. **a**, **b** Platelets were treated with either 50 μg/ml TLR4 function blocking antibody or 50 μg/ml IgG2a before LPS stimulation (5 μg/ml) in absence or presence of ADP. TLR4 inhibition in LPS-treated had minimal effect on attenuating the numbers of P-selectin positive platelets (**a**) but significantly decreased P-selectin MFI fold change **b** in the presence or absence of ADP priming. Pretreatment of platelets with LPS-RS before LPS stimulation significantly decreased number of P-selectin postive platelets and MFI fold change only in ADP-primed platelets. First and third quartiles were represented by the lower and upper boundaries, respectively, and the line within the box represents the median. Whiskers represent the range of data. + represents the mean * *p* < 0.05, #Significance between all treatments and isotype controls (*p*<0.05)

To investigate if LPS-mediated alpha granule secretion in platelets is dependent on TLR4, platelets were pre-treated with TLR4 function blocking antibodies. We confirmed that TLR4 inhibition using a function blocking antibody did not interfere with detection of P-selectin expression in either resting or ADP-activated platelets (Fig. 6a, b). However, TLR4 inhibition significantly attenuated P-selectin expression in LPS-treated platelets. P-selectin MFI fold change in LPS-treated platelets was significantly decreased compared to those without TLR4 inhibition (− 0.032 ± 0.023 vs. 0.0017 ± 0.031, *p* = 0.021) or those treated with the isotype control (− 0.032 ± 0.023 vs 0.076 ± 0.031, *p* = 0.004) (Fig. 6a). TLR4 inhibition, however, did not significantly alter the percentage of P-selectin positive platelets in LPS-stimulated platelets (5.75 ± 1.32 vs 6.25 ± 3.93, *p* = 0.66). In ADP-primed platelets, TLR4 inhibition significantly decreased LPS-mediated P-selectin MFI fold change (0.061 ± 0.066 vs. 0.14 ± 0.11, *p* = 0.0127) but not the isotype control (0.12 ± 0.11, *p* = 0.58). (Fig. 6b).

Inhibition of TLR4 with LPS-RS, a TLR4 antagonist, had no attenuating effect on LPS-mediated P-selectin expression in unprimed platelets. However, in the presence of ADP, LPS-RS significantly lowered LPS-mediated P-selectin expression. Compared to vehicle control, TLR4 inhibition by LPS-RS significantly decreased the percentage of P-selectin positive platelets (19.71% ± 10.86 vs 10.19% ± 4.51, *p* = 0.0003) and MFI fold change (0.20 ± 0.17 vs 0.099 ± 0.13, *p* = 0.0049). (Fig. 6c).

## Discussion

To the authors’ knowledge, this is the first study documenting the expression of functional TLR4 in canine platelets. The present study indicates that platelet response to *E.coli* LPS via TLR4 is amplified by the agonists ADP and TxA_2_ in dogs.

The expression of functional TLR4 on canine platelets highlights a highly conserved mechanism of pathogen recognition utilized by many other cell types in mammals [17]. But unlike other immune cells, thrombin and ADP, which are platelet agonists that stimulate platelets in extending platelet plug formation, upregulate the surface expression of platelet TLR4 [18]. Here, we found that TLR4 resides within the cytoplasm and alpha granules in unstimulated platelets, similar to the findings in human platelets. The upregulation of surface TLR4 in thrombin-activated human platelets is secondary to the activation of calpain with subsequent cleavage of myosin-9 resulting in TLR4 trafficking from the alpha-granules to the platelet plasma membrane [19]. The co-expression of TLR4 and P-selectin on the surface of activated canine platelets suggests that TLR4 trafficking to the platelet surface also could be mediated by alpha-granule secretion. This unique mechanism of TLR4 upregulation in platelets highlights the interplay between hemostasis and innate immunity.

Our results indicate a highly variable expression of surface TLR4 among dogs which is augmented once platelets were activated by thrombin or ADP. The significant correlation between TLR4 expression and the degree of LPS-mediated alpha-granule release in the presence of ADP suggests that the upregulation of TLR4 augments platelets’ sensitivity to LPS. We further confirmed this finding by inhibiting TLR4 in ADP-primed platelets. While TLR4 inhibition did not interfere with ADP-induced activation, it abolished the stimulatory effects of LPS indicating that ADP potentiates LPS-mediated platelet activation by upregulating surface TLR4 expression. Dogs with naturally occurring sepsis are found to be in a hypercoagulable state with elevated thrombin generation and overconsumption of endogenous anticoagulants like antithrombin III [20]. It is unknown at this stage if elevated levels of thrombin in sepsis could trigger upregulation of surface TLR4 expression in canine platelets and, thereby, increases platelet response to endotoxins. In people, platelet TLR4 expression is elevated during sepsis and this upregulation is associated with the severity of sepsis-induced thrombocytopenia [21]. The prognostic and diagnostic significance of platelet TLR4 expression in dogs requires further investigations.

We demonstrated that *E.coli* LPS, to a limited extent, activates canine platelets to undergo alpha-granule secretion required for normal thrombus formation. P-selectin, a marker of alpha-granule secretion, is an integral protein of the alpha granule membrane. In accordance with previous studies, we were unable to detect significant elevation in P-selectin expression, and TxA_2_ generation elicited by LPS [16]. Despite finding a significant increase in the numbers of platelets expressing P-selectin, the lack of MFI fold change suggests that LPS had minimal effect on augmenting P-selectin density, a marker of substantial alpha-granule secretion. Another explanation is the variable P-selectin expression on unstimulated platelets potentially due to stress from handling, excitement or in vitro platelet activation from PRP generation (Fig. 3) [22]. This likely could decrease platelet sensitivity to LPS. Dogs that were easily excitable, stressed and difficult to restrain for blood draws were later on excluded from the TLR4 blocking experiments, as evidenced by the lower mean resting P-selectin expression (Fig. 5a, c).

**Fig. 7:**
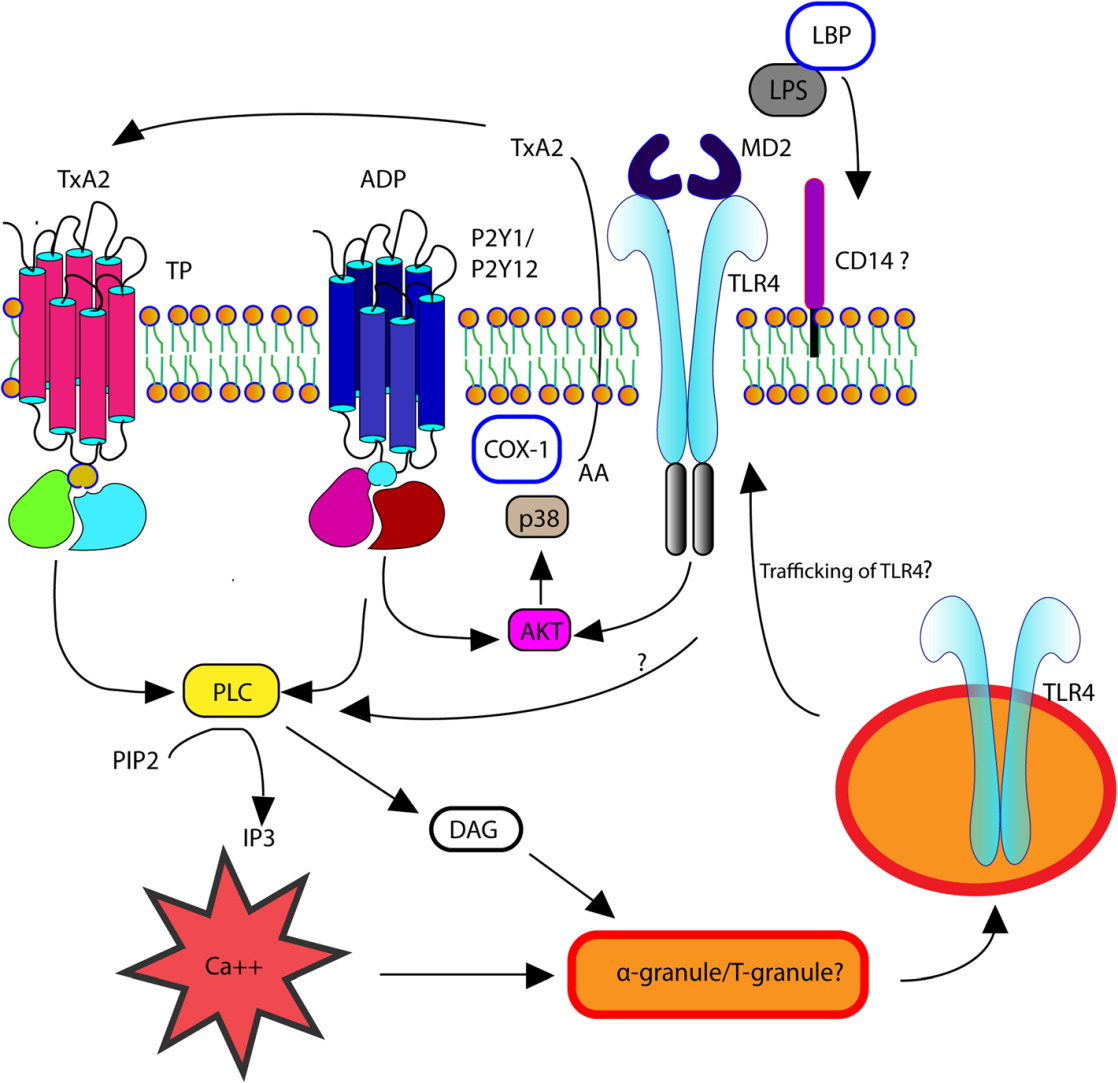
Schematic diagram of LPS-mediated platelet activation and TLR4 expression in canine platelets. ADP activation via P2Y1 or P2Y12 receptor upregulates surface TLR4 expression. TLR4 trafficking to cell membrane from granules may be mediated by alpha-granule secretion. LPS binding protein (LBP) presents LPS to CD14 forming a heterodimeric complex with TLR4 and myeloid differentiation protein 2 (MD-2). Downstream signaling pathway of TLR4 leads to α-granule secretion, which is amplified by ADP and thromboxane A_2_ (TxA_2_). Activation of G-protein coupled receptors, P2Y1/P2Y12 and thromboxane receptor (TP), leads to phospholipase C (PLC) activation and, subsequently, 1,4,5-triphosphate (IP_3_) and diacylglycerol (DAG) for intracellular calcium release and alpha-granule secretion. LPS acts synergistically with ADP to increase generation of TxA_2_, serving a positive feedback mediator. TLR4 and ADP signaling activates cyclooxygenase-1 (COX-1), which converts arachidonic acid (AA) to TxA_2_, likely by the Akt/p38 MAPK pathway

Once platelets are primed with ADP, treatment of platelets with LPS potentiates alpha-granule secretion and TxA_2_ generation, suggesting that LPS synergizes with ADP in amplifying platelet response to LPS. This observation might be due to several underlying mechanisms. First, surface P-selectin on human platelets has been shown to enhance *E.coli* LPS binding to platelet TLR4 by forming TLR4/CD62P receptor complex [23]. Secondly, besides upregulating surface TLR4 on platelets, ADP, which binds to the G-protein coupled receptors, P2Y1 and P2Y12, triggers signaling events critical for robust platelet activation to occur. For example, in murine and human platelets, P2Y1, which couples with G_q_, activates phospholipase C (PLC) and, subsequently, 1,4,5-triphosphate (IP_3_) and diacylglycerol, to increase calcium mobilization and cytosolic calcium concentration, an event required to trigger numerous cellular events including integrin activation. Calcium mobilization via PLCγ2 also is required for TLR4 signalling in immune cells like macrophages and dendritic cells [24]. Whether calcium flux orchestrated by ADP-mediated signaling amplifies TLR4 downstream signaling in canine platelets requires further investigation. In addition to alpha-granule secretion, we also found that LPS and ADP synergistically increase production of TxA_2_, an eicosanoid converted from arachidonic acid within the cell membrane by the enzyme, cyclooxygenase-1. This suggests that TLR4 and ADP receptor signaling share a similar downstream pathway that results in TxA_2_ generation. Nocella et al. proposed that activation of TLR4 and ADP receptors results in amplification of the AKT-p38MAP kinase axis leading to phospholipase A2 phosphorylation and subsequent generation of TxA_2_ in human platelets [16]. Further studies are needed to characterize the non-genomic pathway of TLR4 in dog platelets (Fig. 7).

By directly inhibiting the enzyme, cyclo-oxygenase (COX-1) with ASA, we showed that platelet activation mediated by TLR4 and ADP signaling is partially dependent on TxA_2_. Given its short half-life (~ 30 s), TxA_2_ acts as an autocrine or paracrine to nearby platelets ampliflying platelet activation via platelet TxA_2_ receptors. As expected, ASA did not completely attenuate LPS-mediated alpha granule secretion and, in some dogs, had no effect on P-selectin expression. This may be due to the variable thromboxane responsiveness found in some dogs, in which inhibition of TxA_2_ production may not negatively impact LPS-mediated platelet activation [25]. This also may be due to the presence of other mediators likely involved in the positive feedback mechanism of TLR4 signaling. Antiplatelet therapy is the cornerstone of antithrombotic therapy in dogs. In addition to their antithrombotic properties, antiplatelet drugs have been shown to modulate inflammation in people and dogs by reducing acute phase response and proinflammatory biomarkers like C-reactive protein [26]. Since TxA_2_ serves as a positive feedback in amplifying LPS-mediated platelet activation, antiplatelet therapy may have potential benefits in clinical septic dogs [26]. In observational studies, prehospital administration of ASA has been shown to be associated with reduced mortality and lower prevalence of acute respiratory distress syndrome in critically ill humans with sepsis [27, 28]. Prospective clinical trials are needed to identify the clinical benefits of ASA therapy in at-risk dogs.

The present study has several limitations. First, the concentration of LPS that consistently activated canine platelets in the absence or presence of ADP was 5 μg/ml, a higher concentration than expected in systemic circulation during sepsis. A plausible explanation for the need of this concentration is the low levels of plasma proteins in our washed platelet system. The binding of LPS to TLR4 is complex and requires 3 other extracellular proteins including LPS binding protein (LBP), CD14, and myeloid differentiation protein 2 (MD-2). LBP, a soluble acute-phase protein, binds to LPS and presents it to CD14 on platelets to form a heterodimeric complex with TLR4 and its accessory protein, MD2 (Fig. 6). Whether human platelets express CD14 is controversial [13, 29, 30]. Damien et al. found that the response to LPS in washed platelets is dependent on soluble CD14 suggesting that platelets may obtain CD14 from systemic circulation [30]. We supplemented canine platelets with a small concentration of canine serum in order to enhance platelet response to LPS. However, we did not investigate if canine platelets express the necessary TLR4 signaling complex including surface CD14 expression. Since LPS-RS antagonizes LPS by directly competing with LPS for the binding site on MD-2, our data suggests that dog platelets may express MD-2 on the cell surface. Further studies using platelet rich plasma to determine if concentrations of LPS found in septic dogs could activate platelets is needed. Another plausible explanation is that canine platelets may have variable responses to LPS from different strains of *E. coli* as the affinity for TLR4 on mouse platelets is dependent on the LPS serotypes [23]. Finally, we did not investigate the effects of LPS on other markers of platelet activation such as fibrinogen binding, dense granule secretion and CD40L expression, which have all been shown to increase by LPS stimulation in human and murine platelets [13, 23].

## Conclusion

Our study demonstrated that canine platelets express functional TLR4, which can be upregulated by thrombin or ADP. Although *E coli* LPS is a limited stimulus for platelet activation, in the presence of ADP or TxA_2_, LPS is a potential platelet activator in dogs. The findings of this study provide novel insights into the mechanisms of thrombosis and potential therapeutic targets in septic dogs.

## Methods

### Animals

The study protocol was approved by the Institutional Animal Care and Use Committee at the University of California, Davis. Thirty clinically healthy staff- or student-owned dogs greater than 5 kg were used in this study and 8 dogs were enrolled for each experiment. Dogs were deemed to be clinically healthy by physical examination performed by the corresponding author and a complete blood count using an automated hematology analyzer (Coulter ACT diff, Beckman-Coulter Inc., Miami, FL) and blood smear evaluation. Dogs were not enrolled in the study if they were vaccinated 30 days prior to enrollment, on any concurrent medications, or had any abnormalities on hematological examination. Eight dogs were enrolled.

### Generation of gel-filtered platelets

Whole blood (4 to 6 ml) was drawn from either the jugular or cephalic vein using a 22 gauge needle connected to a 6 ml syringe before transferring into 3.2% sodium citrate tubes. Blood tubes were gently inverted 2 to 3 times and carefully inspected for clots. Citrated blood was transferred to polypropylene tubes and platelet rich plasma was generated by centrifugation (300 x *g,* 5 min, no brakes) at room temperature. Platelets were separated from plasma by gel-filtration over a Sephrose 2B column at 37 °C and eluted with filtered Tyrodes-HEPES buffer (pH 7.4, 5 mM dextrose, 0.5% canine serum, without divalent cations) [31]. Gel-filtered platelets were observed to determine if they exhibited the “swirling” characteristic found in truly discoid resting platelets [31, 32]. Isolated platelets that failed to display swirling movements were not included in the study. Platelet count was obtained using an automated analyzer (Coulter ACT diff, Beckman-Coulter Inc., Miami, FL) and confirmed by bloodsmear evaluation. All experiments were carried out in a sterile manner.

### Detection of platelet TLR4 and P-selectin surface expression by flow cytometry

Platelet count was adjusted to a concentration of 1 × 10^7^ cells/ml with Tyrodes-HEPES (pH 7.4, 5 mM dextrose, 0.5% canine serum, without divalent cations) and platelets were either unstimulated (resting) or stimulated with 0.1 unit/ml bovine-derived alpha-thrombin (Haematologic Technologies, Inc., Essex Junction, VT) or ADP (1 μM or 10 μM) for 15 min at 37 °C [33]. Surface TLR4 expression was assessed using a biotinylated mouse anti-human monoclonal antibody to TLR4 (1:400, Clone: HTA 125, BioRad Laboratoires, Herculues, CA), known to cross react with canine TLR4 [34]. Following 45 min of incubation at 37 °C, platelets were incubated with streptavidin conjugated to phycoerythrin cy7 (1:200, Invitrogen, Carlsbad, CA) for an additional 45 min at 37 °C. For the detection of P-selectin (CD62p), platelets were either resting or first primed with 10 μM ADP (15 min, 37 °C) prior to treatment with 1, 5, or 10 μg/ml LPS from *Escherichia coli* 0111:B4 (EMD Millipore, Temecula, CA) for 30 min at 37 °C. Unstimulated and thrombin-activated (0.1 U/ml) platelets served as negative and positive controls, respectively. Following activation, platelets were incubated with fluorescein isothiocyanate-conjugated rat anti-mouse monoclonal antibody to P-selectin (CD62p) (1:200, Clone:RB40.34, BD Biosciences, San Diego, CA) for 45 min at 37 °C. Platelets were identified by forward and side scatter properties as well as the platelet integrin, β3a (CD61), using a mouse anti-human monoclonal antibody conjugated to allophycocyanin (1:1000, Clone:VI-PL2, eBioscience, San Deigo, CA) (45 min, 37 °C). Cells were fixed in 0.1% paraformaldehyde and analyzed using a 5-color flow cytometer (Beckman-Coulter FC500, Beckman-Coulter Inc., Miami, FL) within 4 h. Gating boundaries were established by fluorescence-minus-one controls. Anti-mouse compensation beads (BD Biosciences, San Diego, CA) conjugated to matched experimental fluorochromes were used for compensation and compensation matrices were calculated using commercially available software (FlowJo, Tree Str Inc., Ashland, OR). TLR4 and P-selectin expression was measured as perecent positive events or fold change in mean fluorescence intensity (MFI) between activated platelets and resting platelets using the following formula:

$$ \mathrm{MFI}\ \mathrm{fold}\ \mathrm{change}=\left({\mathrm{Log}}_{10}\ {\mathrm{MFI}}_{\mathrm{Activated}}\right)-\left({\mathrm{Log}}_{10}\ {\mathrm{MFI}}_{\mathrm{Resting}}\right) $$

The platelet and platelet-derived microvesicle (PDMV) gates were determined as previously described using 0.5 μm and 3 μm calibration beads [35, 36]. In brief, PDMV were quantified based on either the number of CD62P-positive events or MFI fold change in CD62P. Flow cytometry data were analyzed using commercially available software (FlowJo, Tree Str Inc., Ashland, OR).

### Immunofluorescence microscopy of platelet TLR4

Gel-filtered platelets (1 × 10^8^/mL) were either unstimulated or activated with 0.1 unit/ml thrombin or 10 μM ADP. Following activation, platelets were fixed in 1% paraformaldehyde for 15 min at room temperature and concentrated onto microscope slides using a cytocentrifuge (Cytospin 4, ThermoScientific Inc., Grand Island, NY) at 1500 rpm for 5 min. Platelets were then washed 3 times in phosphate buffered saline (pH 7.4) and were either unpermeabilized or permeabilized with 0.1% NP40 (Surfact-AMPs™ NP-40, Pierce, Rockford, IL) for 2 min at room temperature. After washing, cells were blocked with 10% bovine serum albumin (1 h, 37 °C), and subsequently incubated with biotinylated mouse anti-human monoclonal antibody to TLR4 (25 μg/ml, Clone:HTA125, BioRad Laboratories, Hercules, CA) (1 h, 37 °C), followed by streptavidin conjugated to Alexa Fluor 555 (1:200 in 2% BSA, S21381, ThermoFisher Scientific, Waltham, MA) (1 h, 37 °C). P-selectin (CD62P) and integrin β3a (CD61) were detected by incubating cells with rat anti-mouse CD62P monoclonal ab (10 μg/ml, Clone:RB40.34, BD Biosciences, San Diego, CA) and mouse anti-human monoclonal antibody (1 μg/ml, Clone:VI-PL2, eBioscience), respectively, followed by incubation with Alexa Fluor 488-conjugated goat anti-rat IgG and Alexa Fluor 405-conjugated goat anti-mouse IgG (eBioscience, ThermoFisher Scientific, Waltham, MA) overnight at 4 °C. The final dilution of all the secondary antibodies used was 1:50 diluted in 5% goat serum. After washing, a #1.5 glass coverslip was placed on the cells with an anti-fade mounting medium (ProLong Gold, ThermoFisher Scientific, Waltham, MA) and cured overnight at 4 °C. Interfrence controls were prepared by excluding incubation with the primary antibodies in the second immuno-labelling step.

Fluorescent images were acquired using a combination of confocal and super-resolution stimulated emission depletion (STED) microscopy (Leica TCS SP8 STED 3x, Leica Microsystems, Buffalo Grove, IL). Imaging powers of STED wavelengths were set to 20 to 50% of excitation wavelengths. The following imaging sequence was performed to avoid photobleaching: 1. CD61 was first detected by confocal microscopy excited with 405 nm with 1–6 nsec HyD gating; 2. Alexa Fluor 555 (TLR4) was then excited with 555 nm and 660 nm STED depletion laser; 3. Alexa Fluor 488 (P-selectin) was excited with 488 nm and 592 nm STED depletion laser with 1.2–6 nsec HyD gating. All images were acquired at 100x magnification with pixel dwell time of 800 nsec; pixel size (20 to 25 nm) was optimized at four times the image format of 512 × 512 pixels. Deconvolution of microscope images was performed using commercially available software (Huygens Professional, 18.10, Sceintific Folume Imaging) and analyzed using publically available software (FIJI, NIH).

### Thromboxane B_2_ ELISA

Resting or ADP-primed platelets (10 μM ADP,15 min, 37 °C) were treated with 5 μg/ml *E.coli* 0111:B4 LPS (EMD Millipore, Temecula, CA) for 30 min at 37 °C. Platelets pre-treated with 100 μM acetylsalicytic acid (ASA) (Sigma-Aldrich, St. Louis, MO) (30 min, 37 °C) served as negative control. Immediately following stimulation, samples were centrifuged (1500 RPM, 18 °C, 10 min, no brakes) and platelet supernatant was collected, flash frozen in liquid nitrogen and stored at − 80 °C until analysis. Concentration of thromboxane B_2_ (TxB_2_) in the platelet supernatant was measured using a commercial ELISA kit (Enzo Life Sciences Inc., Farmingdale, NY).

### Platelet TLR4 inhibition

Gel-filtered platelets, corrected to 1 × 10^7^ cells/ml with Tyrodes-HEPES, were either incubated with preservative-free function blocking antibody to TLR4 (50 μg/ml, Clone:HTA125, BioRad Laboratories, Hercules, CA) or equivalent concentration of mouse IgG2a (Clone: OX-34Mouse IgG2a, BioRad Laboratories, Hercules, CA) as isotype control for 20 min at room temperature. An additional population of platelets were incubated with 50 μg/ml ultrapure LPS from *Rhodobacter sphaeroides* (LPS-RS) (InvivoGen, San Diego, CA), a TLR4 antagonist, for 1 h at 37 °C. Following TLR4 blocking, platelets were either unstimulated (resting) or stimulated with 10 μM ADP (15 min, 37 °C) before treatment with 5 μg/ml LPS (30 min, 37 °C). CD62P and CD61 were labeled as described above in room temperature.

### Inhibition of platelet cyclooxygenase-1

Platelets (1 × 10^7^ cells/ml) were first treated with 100 μM ASA or an equal volume of deionized water as vehicle control, for 30 min at 37 °C. Resting or ADP-stimulated (10 μM, 15 min) platelets were then treated with 5 μg/ml LPS for 30 min at 37 °C. Surface P-selectin and CD61 were labeled and detected by flow cytometry as described above.

### Statistical analysis

Normality of data was tested using Shapiro-Wilk normality test or visual inspection of normal quartile plots. Non-parametric data were presented as median and interquartile range (IQR) and parametric data were presented as mean ± standard deviation. Normally distributed and paired data were analyzed using t-tests while nonparametric and paired data were analyzed using Wilcoxon signed-rank test. Unpaired data was measured using either Mann-Whitney U test or unpaired t test. One-way repeated measures ANOVA was used for comparing the means of dose-response studies followed by post-hoc analysis using Dunnet test. Data that violated the assumption of sphericity were analyzed using Greenhouse-Geisser correction. Correlation and correlation coefficiency were calculated using Pearson correlation. An alpha-priori of < 0.05 was considered statistically significant. Interindividual variability was calculated as the ratio between the standard deviation of a group and its means and expressed as coefficient of variation (CV). Data were analyzed using commercially available software (Prism 7.0,GraphPad Software, La Jolla, CA).

## Data Availability

The datasets used and/or analyzed during the current study are available from the corresponding author on reasonable request.

## Abbreviations

ASA: Acetylsalicylic acid
LPS-RS: LPS from *Rhodobacter sphaeroides*
MFI: Mean fluorescence intensity
TLR4: Toll-like receptor 4
TxA_2_: Thromboxane A_2_
TxB_2_: Thromboxane B_2_
